# Operational Limits of the Bulk Hybrid Liquid Membranes Based on Dispersion Systems

**DOI:** 10.3390/membranes12020190

**Published:** 2022-02-05

**Authors:** Andreea Ferencz (Dinu), Alexandra Raluca Grosu, Hussam Nadum Abdalraheem Al-Ani, Aurelia Cristina Nechifor, Szidonia-Katalin Tanczos, Paul Constantin Albu, Mihaela Emanuela Crăciun, Mihail-Răzvan Ioan, Vlad-Alexandru Grosu, Gheorghe Nechifor

**Affiliations:** 1 Analytical Chemistry and Environmental Engineering Department, University Politehnica of Bucharest, 011061 Bucharest, Romania; andra_d24@yahoo.com (A.F.); andra.grosu@upb.ro (A.R.G.); hussamalani32@gmail.com (H.N.A.A.-A.); me.craciun@gmail.com (M.E.C.); ghnechifor@gmail.com (G.N.); 2 Chemical Industries Department, Institute of Technology, Middle Technical University, Al Zafaraniyah, Baghdad 10074, Iraq; 3 Department of Bioengineering, University Sapientia of Miercurea-Ciuc, 500104 Miercurea-Ciuc, Romania; tczszidonia@yahoo.com; 4 Radioisotopes and Radiation Metrology Department (DRMR), IFIN Horia Hulubei, 023465 Măgurele, Romania; paulalbu@gmail.com (P.C.A.); razvan.ioan@nipne.ro (M.-R.I.); 5 Department of Electronic Technology and Reliability, Faculty of Electronics, Telecommunications and Information Technology, University Politehnica of Bucharest, 061071 Bucharest, Romania

**Keywords:** liquid membranes, hybrid design liquid membranes, operational parameters, silver ion transport, p-nitrophenol transports, membrane flux, membrane selectivity, membrane system stability

## Abstract

Liquid membranes usually have three main constructive variants: bulk liquid membranes (BLM), supported liquid membranes (SLM) and emulsion liquid membranes (ELM). Designing hybrid variants is very topical, with the main purpose of increasing the flow of substance through the membrane but also of improving the selectivity. This paper presents the operational limits of some kind of hybrid membrane constituted as a bulk liquid membrane (BLM), but which works by dispersing the aqueous source (SP) and receiving (RP) phases, with the membrane itself being a dispersion of nanoparticles in an organic solvent (NP–OSM). The approached operational parameters were the volume of phases of the hybrid membrane system, the thickness of the liquid membrane, the working temperature, the flow of aqueous phases, the droplet size of the aqueous phases dispersed across the membrane, the nature and concentration of nanoparticles in the membrane, the pH difference between the aqueous phases, the nature of the organic solvent, the salt concentration in the aqueous phases and the nature of transported chemical species. For this study, silver ion (SI) and *p*-nitrophenol (PNP) were chosen as transportable chemical species, the *n*-aliphatic alcohols (C_6_…C_12_) as membrane organic solvents, 10–undecenoic acid (UDAc) and 10-undecylenic alcohol (UDAl) as carriers and magnetic iron oxides as nanoparticles dispersed in the membrane phase. Under the experimentally established operating conditions, separation efficiencies of over 90% were obtained for both ionic and molecular chemical species (silver ions and *p*-nitrophenol). The results showed the possibility of increasing the flow of transported chemical species by almost 10 times for the silver ion and approximately 100 times for *p*-nitrophenol, through the appropriate choice of operational parameters, but they also exposed their limits in relation to the stability of the membrane system.

## 1. Introduction

Liquid membranes represent a constantly expanding field of research for both analytical and applied separatology, justified by the excellent performance in selectivity [1,2], separation efficiency [3,4] and technical–economic accessibility [5,6]. This is because a liquid membrane is generally any barrier of a liquid nature (homogeneous or heterogeneous, hydrophilic or hydrophobic or emulsified or deposited on supports) interposed between two fluids, with the ability to selectively block the passage from one fluid to another of some or several components [7,8].

Liquid membranes (LM) separation systems consist of two homogenous liquid phases, immiscible with the membrane, denoted as the source phase (SP) and receiving phase (RP). The separation of the two liquid phases is achieved with a third liquid, the membrane (M), which acts as a semi-permeable barrier between the two liquid phases [7,8,9,10].

**Figure 1:**
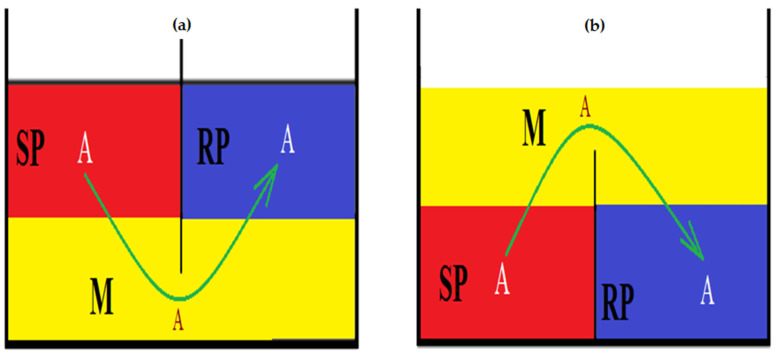
Schematic representation of membrane systems with an organic solvent: (**a**) denser or (**b**) less dense than the aqueous phases. Legend: M = membrane; SP = Source Phase; RP = Receiving Phase; A = interest chemical species for separation.

A well-established graphic and practical conception of liquid membranes (Figure 1) takes into account the density of the membrane, which is generally an organic solvent or a multicomponent system, in which the continuous phase is the organic solvent [11,12].

The density of the membrane phase becomes unimportant if the membrane solvent is immobilized in or on a support [12,13,14,15], thus obtaining the supported liquid membranes (SLM). An interesting variant, but not yet sufficiently evaluated in separation processes, is a liquid membrane based on a magnetic liquid (ferrofluid) [16]. Furthermore, this solution has no restrictions on the density of the organic solvent but involves special aspects in stability and mass transfer [17].

Due to the research intensification, the applications of the liquid membrane system have been extended to a multitude of fields, such as wastewater treatment; analytical, inorganic and organic chemistry; chemical engineering and biomedical and biotechnological engineering. Thus, research in these fields has developed a number of applications of liquid membranes: removal of organic compounds, recovery of fermentation products, gas separation and recovery of precious or toxic metals [18,19,20,21,22,23].

Given the large number of developed applications, bulk liquid membranes (BLM) have remained extremely interesting for laboratory testing of the performance of various solvents and/or carriers, being designed and built in most variants. Thus, the cylindrical membrane system is known, with a solid, either flat [24] or cylindrical, barrier [24,25] separating the aqueous phases. Burgard et al. also proposed a change to the classical cell model by adding a rotating cylinder [26].

The cells of the membrane system have more and more diversified geometries, like a tube in the shape of H, V, W or U, a tube in a tube, semi-circular enclosures, etc. These can be provided with various stirring systems: mechanical, magnetic, with static turbulence promoters, etc., with the stirring systems having the purpose of reducing the resistance that normally occurs during mass transfer. In such membrane cells, the SP and RP are often formed of polar liquids (aqueous solutions or a mixture of solvents), with these being separated by membrane M (Figure 1), immiscible with the two polar phases [24,25,26,27,28,29,30].

In the last years, technologies similar to MLV have been discovered and developed, all based on the same principle: extraction through an undispersed selective membrane, coupled with perm-selective diffusion of solute-extracting complexes and redissolution of the solute in a continuous dynamic process. Among the membranes that are part of the BLM domain, we can list hybrid liquid membranes (HLM) [31], liquid membranes thin capillary or tubular fibers (HFCLM) [32], hollow fiber liquid membranes (HFLM) [33], hybrid multi-membrane systems (HMS) [34], membrane contactor systems [35,36,37] and membranes based on extraction and redissolution [38,39].

Previously studied in a silver ion separation process based on dispersed systems [40], a hybrid liquid membrane allows the variation of an important number of constructive and operational parameters.

This paper presents the operating limits of this type of hybrid membrane constituted as a bulk liquid membrane (BLM), but which works with the dispersion of the aqueous source phase (SP) and the receiving phase (RP). The membrane is a dispersion of magnetic nanoparticles in an organic solvent (NP–OSM).

The target chemical species, silver ions (SI) and *p*-nitrophenol (PNP), were chosen for their technical–economic importance or environmental impact but also for the availability of comparative data from previous research, which are very useful for the most objective assessment of hybrid system performance [40,41,42,43].

## 2. Materials and Methods

### 2.1. Reagents and Materials

All reagents used in the presented work were of analytical grade. They were purchased from Merck (Merck KGaA, Darmstadt, Germany): silver nitrate, sodium chloride, sodium hydroxide, sodium chloride, sodium nitrate, hydrochloric acid, nitric acid and *p*-nitrophenol.

The membrane components were purchased from Sigma-Aldrich (Merck KGaA, Darmstadt, Germany): *n*-hexanol, *n*-octanol, *n*-decanol, *n*-dodecanol, 10–undecylenic acid (undecenoic acid) and undecylenic alcohol (10–undecen–1–ol or 11–hydroxy–1–undecene), and they have the characteristics presented in Table 1 [44].

The purified water characterized by 18.2 μS/cm conductivity was obtained with an RO Millipore system (MilliQ^®^ Direct 8 RO Water Purification System, Merck, Darmstadt, Germany).

The iron-based magnetic nanoparticles were obtained by the electrochemical method, previously presented in detail [40,41,42]. In this case, the electrolysis with iron electrodes was performed in pure water (to obtain magnetic nanoparticles of iron oxides) and in a silver nitrate electrolyte of 10^−1^ mol/L (to obtain magnetic nanoparticles based on silver and iron oxides).

The obtained magnetic nanoparticles’ characteristics are presented in Table 2.

### 2.2. Methods

#### 2.2.1. Obtaining the Dispersion Liquid Membranes (DLM)

The membrane dispersions were achieved by dispersing 1–3 g of magnetic nanoparticles into the considered aliphatic *n*-alcohol, which contained 10^−3^ mol/L of 10–undecylenic acid (UDAc) or 10–undecen–1–ol (UDAl). A volume of 600 mL (about 498 g) of *n*-alcohol (*n*-decanol) and 2.0 g of iron or silver-iron oxide nanoparticles were placed in an 800 mL glass tank. In this system, 1 g of 10–undecen–1–ol was then added. In another 800 mL vessel, the same components were introduced, and then 1g of 10–undecylenic acid was added.

The dispersion of magnetic nanoparticles was performed in the ultrasound field for 4 h, using the Elmasonic S ultrasonic bath from Elma Schmidbauer (Elma Schmidbauer GmbH, Singen, Germany). Finally, a dark brown dispersed liquid system was formed. The dirty yellow opalescent dispersion had a homogeneous behaviour without changes of appearance (aggregations, sedimentation and color changes) for at least seven days.

#### 2.2.2. Transport Experiments of the Silver Ions or *p*-Nitrophenol

**Figure 2:**
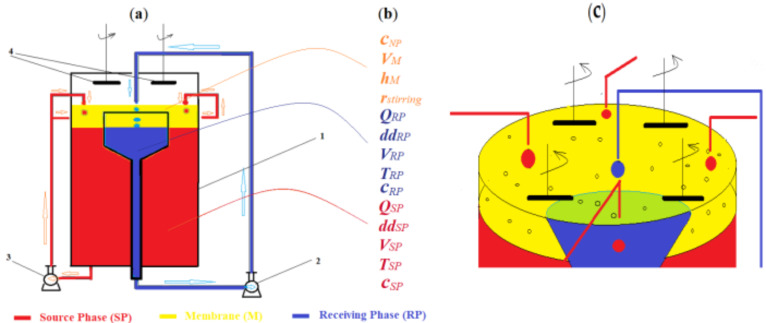
The dispersion bulk liquid membrane module: (**a**) functional scheme: 1—the module; 2 and 3—variable flow pumps; 4—magnetic rod stirrers; (**b**) variable parameters: ***C**_NP_*—nanoparticles’ concentration; ***V**_M_*—membrane volume; ***h**_M_*—membrane height; ***r**_stirring_*—stirring rate; ***Q**_RP_*—receiving phase flow; ***V**_RP_* –receiving phase volume; ***dd**_RP_*—receiving phase drops dimension; ***T**_RP_*—receiving phase temperature; ***C**_RP_*—receiving phase concentration; ***Q**_SP_*—source phase flow; ***V**_SP_*—source phase volume; ***dd**_SP_*—source phase drops dimension; ***T**_SP_*—source phase temperature; ***c**_SP_*—source phase concentration; (**c**) positioning of drip holes and magnetic rods.

The study of the transport of the two target chemical species, silver ion and *p*-nitrophenol, was performed using an installation of our own design (Figure 2a), previously presented in detail [40]. The target chemical species were dissolved in pure water to form two stock solutions, with a concentration of 10^−2^ mol/L.

The concentration of the studied chemical species and their pH were obtained using nitric acid for the silver ion and hydrochloric acid for *p*-nitrophenol. The adjustment was completed with sodium hydroxide solution of 10^−1^ mol/L, freshly prepared.

**Figure 3:**
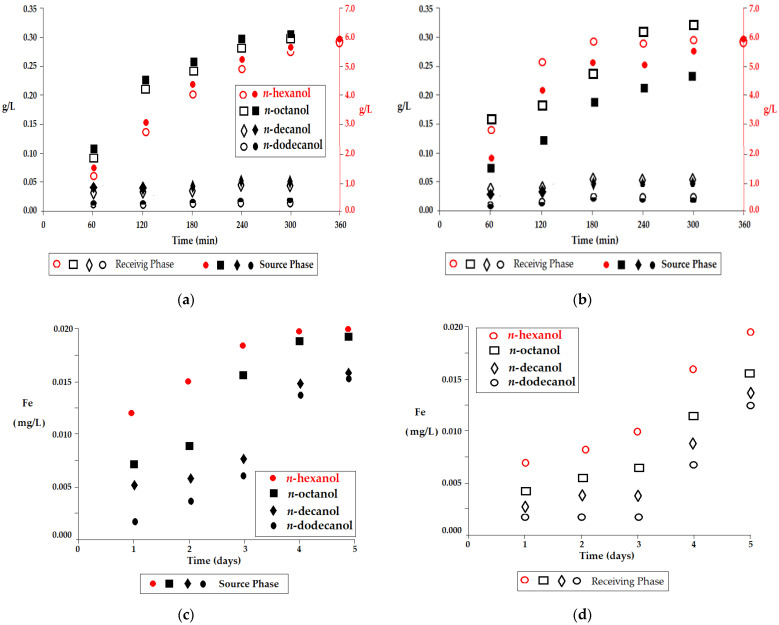
Material losses from the dispersion bulk liquid membrane: (**a**) *n*-alkyl alcohol in the aqueous phase (pH = 7 in the source phase, and pH = 1 in the receiving phase); (**b**) *n*-alkyl alcohol in aqueous phase (pH = 1 in the source phase, and pH = 11 in the receiving phase); (**c**) iron in the source phase; (**d**) iron in the receiving phase.

The influence of the operational parameters of pertraction (Figure 2b) was obtained using the pertraction module (Figure 2a), which allowed the variation of both physical parameters (ionic strength and temperature) and chemical ones (nature and concentration of chemical species of interest and environmental pH) of the study. The dispersion of the source and receiving phase (Figure 3c) was done by a drip, adjusting the flow from a four-way peristaltic pump (for the source phase) and a one-way peristaltic pump (for the receiving phase). The liquid membrane based on the dispersion of magnetic nanoparticles was kept in suspension, by means of four magnetic rods, and placed 5 mm from the membrane surface, by concurrent rotation with a variable speed electric motor.

Monitoring of the concentration of the chemical species in the membrane phases was performed by ultraviolet and visible spectrometry (UV-Vis) methods, and it was validated by atomic absorption spectrometry (AAS) for silver and iron ions and by ultraviolet and visible spectrometry (UV–Vis) for *p*-nitrophenol and *n*-alcohols. *p*-nitrophenol determinations were validated by electrochemical amperometry with a specific biosensor.

In all determinations, the principles of quality assurance of the chemical measurements recommended by the EURACHEM guide were observed [45,46].

The extraction efficiency (EE %) for the silver ions or *p*-nitrophenol was calculated as follows [47,48], based on the solution concentration:

(1) $$EE\left(\%\right)=\frac{\left(c_{0}-c_{f}\right)}{c_{0}}\cdot 100$$

with *c_f_* being the final concentration of the solute (silver ions or *p*-nitrophenol) and *c*_0_ being the initial concentration of the solute (silver ions or *p*-nitrophenol).

The same extraction efficiency could also be obtained based directly upon the absorbance of the considered solutions (silver ions or *p*-nitrophenol) [49,50], as in:

(2) $$EE\left(\%\right)=\frac{\left(A_{0}-A_{s}\right)}{A_{0}}\cdot 100$$

with *A*_0_ being the initial absorbance of the sample solution and *A_s_* being the current absorbance of the sample. The experiments were performed at least three times, in the same working conditions, and each time, the number of collected samples was three (of 1 mL each).

### 2.3. Equipment

To assess and validate the content in the metal ions of the samples, the atomic absorption spectrometer AAnalyst 400 AA Spectrometer was used (Perkin Elmer Inc., Waltham, MA, USA), with a single element hollow-cathode lamp, using WinLab32—AA software (Perkin Elmer Inc., Waltham, MA, USA). For determination of the iron content, the operating current was set at 2 mA and the wavelength at 248.3 nm, with a spectral bandwidth of 0.2 nm. For silver, the values of the experimental parameters were a wavelength of 328.1 nm, a spectral bandwidth of 0.7 nm, and an operating current of 5 mA.

The UV–Vis studies on the nanoparticles’ component samples (silver and/or iron) were performed on dual-beam UV equipment–Varian Cary 50 (Agilent Technologies Inc., Santa Clara, CA, USA) at a resolution of 1 nm, spectral bandwidth of 1.5 nm, and a scan rate of 300 nm/s. The UV–Vis spectra of the samples were recorded for a wavelength ranging from 200 to 800 nm, at room temperature, using 10 mm quartz cells.

Furthermore, the UV–Vis validation analysis of the silver and iron ions or *p*-nitrophenol solutions was performed on a CamSpec M550 spectrometer (Spectronic CamSpec Ltd., Leeds, UK). The UV–Vis spectra of the samples were recorded for a wavelength ranging from 200 to 800 nm, at room temperature, using 10 mm quartz cells.

The electrochemical analysis was followed up with a PARSTAT 2273 Potentiostat (Princeton Applied Research, AMETEK Inc., Oak Ridge, TN, USA). A setup with a glass cell with three electrodes was used.

The pH, conductance and anions’ concentration (in the source or receiving phase) were determined using a conductance cell or combined selective electrode (HI 4107, Hanna Instruments Ltd., Leighton Buzzard, UK) and a multi-parameter system (HI 5522, Hanna Instruments Ltd., Leighton Buzzard, UK).

## 3. Results and Discussions

The hybrid membrane system, which was the subject of the studies in this paper, was built as a bulk liquid membrane (BLM), but which worked by dispersing the source (SP) and receiving (RP) aqueous phases, and the membrane was a dispersion of nanoparticles in an organic solvent (NP–OSM).

This hybrid system was expected to have the common performances of the bulk liquid membranes (BLM) and emulsion liquid membranes (ELM) but also their disadvantages and operational problems (Table 3).

Starting with the important aspects presented in Table 3, the experimental study on the hybrid system (Figure 2a) was oriented in the direction of determining the favorable working limits for the main parameters, in order to obtain an optimal separation efficiency: the nature of the chemical species in the aqueous phases; working temperature; pH; salt concentration; the volume and recirculation flow of the aqueous phases; the nature, volume and thickness of the membrane and the type and concentration of the nanoparticles and carriers in the membrane (Figure 2b).

In order to determine the influence of the main parameters of the membrane on the transport of silver ion (SI) and *p*-nitrophenol (PNP), we started from the recently presented results [40]. Thus, a volume of the source phase of 5000 mL of a neutral or acidic (made with nitric acid) pH solution was considered, and a volume of the receiving phase of 500 mL of a solution of an acidic pH (made with hydrochloric acid) or a basic pH (made with sodium hydroxide) was considered.

### 3.1. Membrane Material Losses in the Aqueous Phases of the Hybrid System

**Figure 4:**
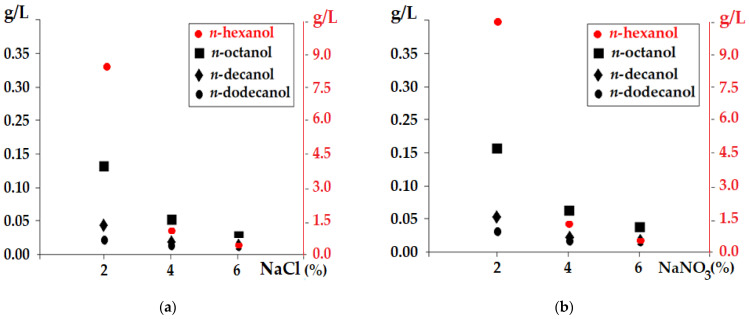
Materials’ loss from the dispersion bulk liquid membrane: (**a**) HCl acid source phase; (**b**) HNO_3_ acid source phase as a function of the weight concentration of the considered salts.

**Figure 5:**
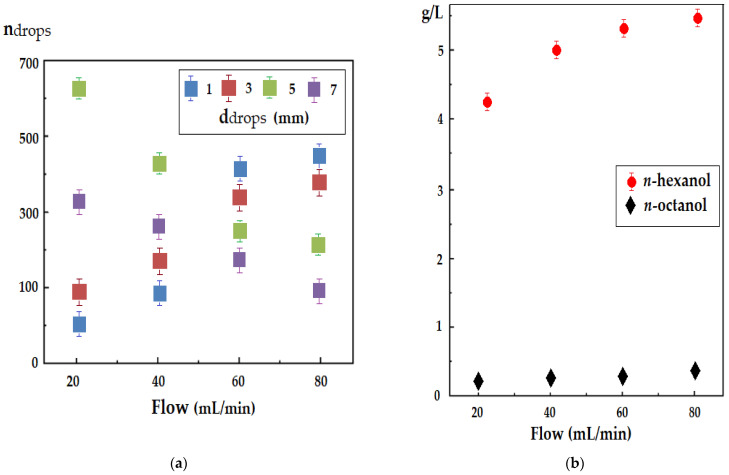
Droplet size depending on flow rate (**a**) and loss of membrane material in the source phase depending on the flow rate for *n*-hexanol and *n*-octanol (**b**).

The liquid membrane was made from *n*-alkyl alcohols (*n*-hexanol, *n*-octanol, *n*-decanol, *n*-dodecanol) as membrane solvents, 10–undecenoic acid (UDAc) and 10–undecylenyl alcohol (UDAl) as carriers and magnetic iron oxides as dispersed nanoparticles. The variable parameters were pH (Figure 3), the concentration of sodium chloride or sodium nitrate (Figure 4), the flow rate of the aqueous phases and the working temperature (Figure 5).

The loss of membrane solvent in the aqueous phases occurred up until the solubility limit at the working temperature, and this process took place quite quickly, after 4 working hours (Figure 3a,b). The amount and rate of membrane solvent loss were more pronounced when the aqueous receiving phase had a basic medium (pH = 11) (Figure 3b), most likely due to the acidity of the alcohols.

The highest solvent loss was found for *n*-hexanol, which otherwise was the most soluble in pure water (Table 1).

From the data illustrated in Figure 3a,b, it could be established that the process of transfer of any species from the source phase to the receiving phase were accompanied by losses (impurities) of the aqueous phases with the alcohol that constituted the membrane. From this perspective, in order to avoid disturbances in the monitoring of the effective mass transfer of the chemical species about to be separated through the membrane, prior to introduction into the extraction module, saturation of the aqueous phases with alcohol may be recommended. For the same reason, in order to be able to perform the discharge into an emissary, the waters treated by such a process, the membrane solvent must have an even number of carbon atoms, which will make it biodegradable, and the aqueous effluent must be compatible with sewage treatment plants. Of the four alcohols used as membrane phases, *n*-hexanol could be excluded at this stage of the study. However, considering source phases with a high ionic strength, generated by strong electrolytes, which are often found in industrial effluents, *n*-hexanol will be considered for the comparison of the results obtained in the extraction with the other membrane solvents.

The appearance of iron in the aqueous phases is an indicator of the stability of the dispersed alcohol–iron oxide nanoparticles system. Although the amounts of iron in the aqueous phases were very small, this process illustrates the hydration of the nanoparticles and the transition from the membrane phase to the aqueous phases. The acidity of the source phase (pH = 1) led to the increase in the amount of iron taken up (Figure 3c), while in the basic environment, the processes of hydration and the capture of nanoparticles in the receiving phase (pH = 11) (Figure 3d) were slower. From the point of view of the stability of the alcoholic nanodispersion, *n*-dodecanol was the most favorable, with the dispersion being relatively stable for about 3 days. From the point of view of the stability of the membrane nanodispersion (*n*-alcohol–iron oxide nanoparticles) as well, the one based on *n*-hexanol should be avoided, and the one based on *n*-octanol should be used in separation processes which operate for a maximum of 3 days (Figure 3c,d).

Adding salts can be an important way to stabilize the membranes based on *n*-alkyl alcohols (Figure 4). By operating at the maximum flow of aqueous solutions, a decrease in losses of normal alcohols from the membrane to the acid source phases was found. The influence of sodium chloride (NaCl) in the acidic aqueous solution of pH = 1 (HCl) (Figure 4a) was greater than that of sodium nitrate (NaNO_3_) in the acidic pH = 1 solution (HNO_3_) (Figure 4b). What is noteworthy is that at weight concentrations of 6% salt in the source acid phases, *n*-hexanol became interesting as a membrane solvent for future applications. For other alcohols, a salt weight concentration of 2–4% could ensure the stability of the membrane.

The drip flow of the source and receiver phases through the membrane in the hybrid membrane system ensured the control of the interphase contact surface. Decreasing the droplet size as a function of the flow rate (Figure 5a) cannot exceed the limit at which the Archimedean force would equal the gravitational force that ensures that the droplet descends across the membrane. Otherwise, it would compromise the membrane process, as aqueous droplets would float across the membrane. From this point of view, the density of the membrane phase should be a little lower than that of water. In the present study, the selected alcohols had densities almost 20% lower than water, but the difference in density between them was small (Table 1).

The addition of iron oxide nanoparticles in quantities not exceeding 3 g/L did not significantly influence the density of the working membrane alcohol.

Limiting the size of the droplets made it possible to control the flow control using several drip holes, with 2–4 flexible silicone rubber tubes (Figure 2c). The study of droplet sizes according to the flow rate of the aqueous phase was carried out with the help of the system designed and presented previously [51,52]. This system consisted of a video camera connected to a computer that allowed counting of the drops, depending on the diameter imposed by the operator. The results presented in Figure 5a show that when varying the flow of the source phase from 20 to 80 mL/min, a relatively narrow distribution of most of the drops of around 4 mm was obtained. At lower flow rates (20–40 mL/min), the distribution was narrower, with most of the drops being around 4 mm in diameter. At a flow rate of 80 mL/min, the distribution flattened, which required obtaining higher flow rates by multiplying the drip holes.

The losses of alcohols in the source phase depending on the drip flow (Figure 5b) were capped by their solubility but also by the adhesion to the surface of the drops. The most soluble alcohols used (*n*-hexanol and *n*-octanol) were chosen for illustration. However, excess dispersed alcohols (above the solubility limit) in the aqueous phases returned to the membrane by coalescence.

The reduction of alcohol losses in the aqueous phases can be achieved by controlling the temperature of the membrane system, provided that it is performed in a closed module. However, this temperature must be kept in the range of 10–40 °C because there may be effects on the separation efficiency. Thus, the increase in temperature favors the solubilization and diffusion of the target species, but it causes additional losses of the membrane solvent, both in the aqueous phases of the system and by evaporation.

**Figure 6:**
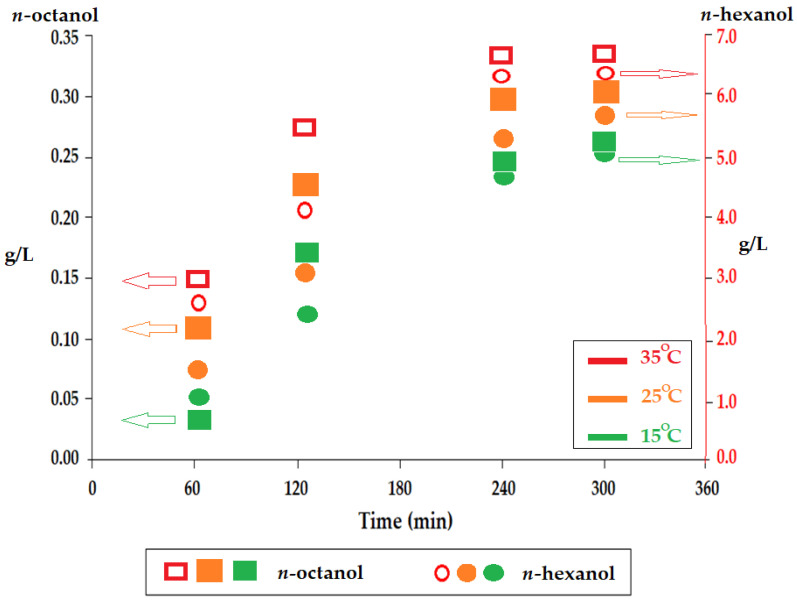
Loss of membrane solvent in the source phase depending on the operating temperature for *n*-octanol and *n*-hexanol.

The losses depending on the temperature (Figure 6) of *n*-hexanol and *n*-octanol, which were the most soluble membrane solvents used in the source phase, were capped by their solubility at the required working temperature. At 15 °C, there were the lowest losses, but also, the adhesion to the surface of the drops could increase because the viscosity increased. From a practical point of view, the operation at 20–25 °C remains a more technically viable working option. The work effort at 15 °C is not justified, with the losses of membrane material not being able to compensate for the energy costs of cooling the system. In applying this technology, it is advisable to work at an ambient temperature, depending on the climatic zone.

### 3.2. The Influence of the Operational Parameters of the Membrane on the Separation Efficiency

To study the efficiency of the operational parameters of the membrane (thickness (*h*), volume of the membrane phase (*V*), solvent nature and concentration of the carriers and magnetic nanoparticles and rotation speed of the magnetic rods), the experiments performed allowed the choice of the following working parameters: 80 mL/min for the flow rate of the source phase (through four drip holes), 20 mL/min for the flow rate of the receiving phase (through only one drip hole), a working temperature of 25 °C, *n*-alkyl alcohols as membrane solvents (*n*-hexanol, *n*-octanol, *n*-decanol and *n*-dodecanol) in case of using at least 4% of the electrolyte in the aqueous phases and a neutral or acidic pH in the source phase and an acidic or base pH in the receiving phase. The choice of the target chemical species, silver ions (SI) and p-nitrophenol (PNP), for the experiments in this membrane system followed the study of both an ionic substance and a molecular one. The primary purpose was to evaluate the effect of convection, by dispersing the aqueous phases across the membrane. The concentration of the target chemical species was chosen so that this parameter ensured maximum extraction efficiency [53,54,55]. It is known that as the concentration of the analyte in the source phase increases, the efficiency of membrane extraction, in a single step, decreases.

**Figure 7:**
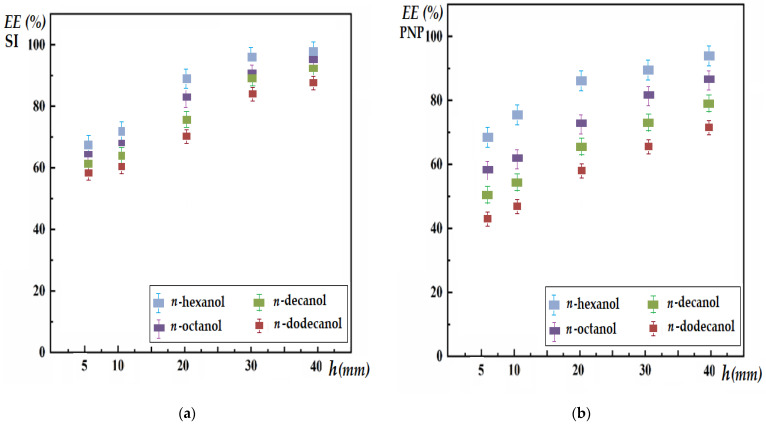
The efficiency of the separation after 3 h of silver ions (SI) and *p*-nitrophenol (PNP), depending on membrane thickness, rotation speed of the magnetic rods and the nature of the solvent: (**a**) membrane thickness at the extraction of SI; (**b**) membrane thickness at the extraction of PNP.

Based on the observations of N.N. Li, who studied the separation of hydrocarbons with a surfactant membrane and emphasized the importance of the size of the ascending drop but also of the path travelled by a layer of hydrocarbons [56,57], it was possible to study the influence of the membrane thickness (*h*) and, implicitly, of the volume (*V*) of the membrane solvent on the efficiency of the separation of silver ions (SI) and *p*-nitrophenol (PNP) (Figure 7), at 3 h of operation under the following working conditions:

- For the extraction of silver ions (Figure 7a,c): a source phase of pH = 7 and a concentration of 10^−4^ mol/L (0.0108 g Ag/L), a receiving phase of pH = 1 (hydrochloric acid), a 4% NaCl electrolyte in the aqueous phases and a membrane of *n*-alkyl alcohols with 3 g/L of magnetic nanoparticles.
- For the extraction of *p*-nitrophenol (PNP) (Figure 7b,d): a source phase with pH = 1 (nitric acid) and a concentration of 10^−2^ mol/L (1.39 g PNP/L), a receiving phase with pH = 11 (sodium hydroxide), a 4% NaNO_3_ electrolyte in the aqueous phases and a membrane of *n*-alkyl alcohols with 3 g/L of magnetic nanoparticles.

The obtained results show that for silver ions (SI), the extraction efficiency depends significantly on the nature of the alcohol and the thickness of the membrane (Figure 7a). A high membrane thickness favors the convection generated by the falling aqueous phase droplets, leading to higher extraction efficiencies for all membrane alcohols. As the thickness of the membrane decreases, the path travelled by the aqueous drop becomes smaller, and the convection that it generates has a minor contribution to the extraction efficiency. For the efficiency of *p*-nitrophenol (PNP) extraction (Figure 7b), the greatest influence is the nature of the alcohol, while the thickness of the membrane has a lower contribution to *n*-hexanol, which increases significantly for higher alcohols. Practically, in this case, the solubility of PNP in the membrane is decisive, but a greater thickness of the membrane amplifies the convection generated by the droplets. From an applicative point of view, a greater thickness of the membrane is recommended, which will force the convection and, implicitly, the extraction efficiency, but the limitation of the concomitant increase of the membrane volume must be taken into account.

For membrane extraction, the experiments suggested the use of *n*-octanol and *n*-decanol for membrane extraction at a membrane thickness of at least 30 mm. Although *n*-hexanol had the best results, the instability of the membrane makes it less competitive. On the other hand, *n*-dodecanol showed the lowest performances, which were determined both by the higher viscosity (Table 1) and by the lower solubility of the target species in the membrane.

**Figure 8:**
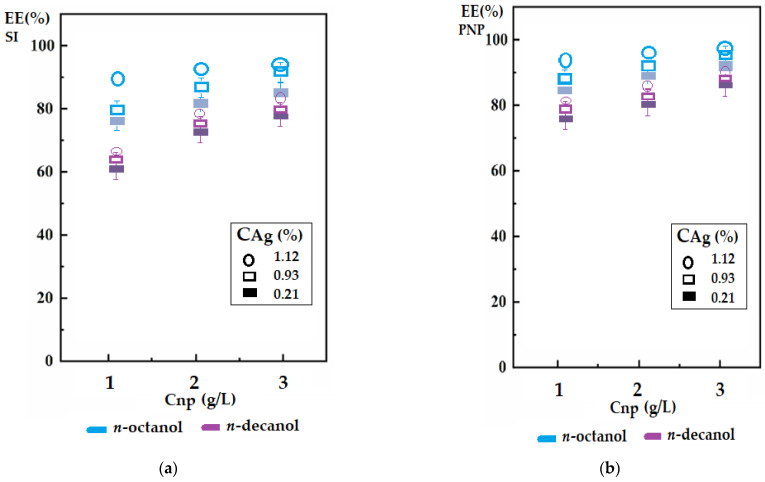
The efficiency of the separation of silver ions (SI) and *p*-nitrophenol (PNP) as a function of the concentration of magnetic nanoparticles (C_np_) in the membrane solvent and their silver content (C_Ag_): (**a**) SI extraction; (**b**) PNP extraction.

In order to observe the influence of the concentration of magnetic nanoparticles and the contribution of silver contained in them on the separation of the target chemical species, the study continued with the investigation of *n*-octanol and *n*-decanol as selected membrane solvents (Figure 8). At a rotational speed of the magnetic rods of 150 rot/min and a membrane thickness of 10 mm, the efficiency of silver extraction (Figure 8a) and *p*-nitrophenol extraction (Figure 8b) were determined. For both test chemical species, the increase of the nanoparticle concentration in the membrane phase but also of the silver concentration in the nanoparticle composition favored the process. At an operating time of 3 h, the data in Figure 8 show that the extraction efficiency of *p*-nitrophenol was superior to that of silver ions, for both *n*-decanol and, especially, for *n*-octanol. The favorable effect of the concentration of nanoparticles in the membrane environment could be attributed primarily to the convection it generated under the action of the rotating magnetic field given by the magnetic rods. It is very interesting to note that their silver content also favored extraction, meaning that the nanoparticles also act as carriers [41].

The experiments performed with larger membrane thicknesses indicated similar influences, but which decreased with an increasing membrane thickness. The effect of magnetic convection could be validated at thicknesses of 5 and 10 mm, while at thicknesses greater than 20 mm of the membrane layer, the results were very minorly affected, being able to fall into experimental and analysis errors.

**Figure 9:**
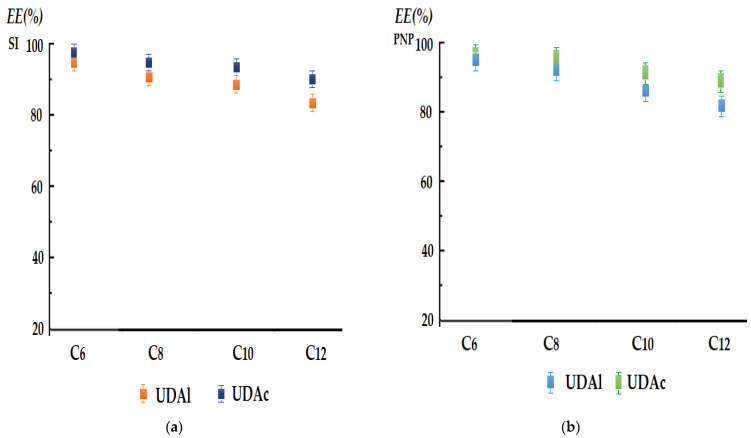
Separation efficiency of silver ions (SI) (**a**) and of *p*-nitrophenol (PNP) (**b**), depending on the concentration and nature of the membrane phase carriers, 10–undecenoic acid (UDAc) and 10–undecylenyl alcool (UDAl).

The membrane composition of the hybrid system also included the carriers, which, in our case, were 10–undecenoic acid (UDAc) and 10–undecylenyl alcohol (UDAl). Their concentrations in the membrane solvent were chosen at 10^−3^ mol/L, in order to ensure the stabilization of magnetic nanoparticles in the dispersed system, as well as to contribute to the transport of target chemical species, especially in the case of silver ions (SI). The efficiency of the extraction of the target chemical species was carried out in the most favorable experimental conditions, as previously determined:

- For the extraction of silver ions (Figure 9a): a source phase of pH = 7 and a concentration of 10^−4^ mol/L (0.0108 g Ag/L), a receiving phase of pH = 1 (hydrochloric acid), a 4% NaCl electrolyte in aqueous phases and a membrane of *n*-alkyl alcohols with 3 g/L of magnetic nanoparticles having a 1.12% silver content.
- For the extraction of *p*-nitrophenol (PNP) (Figure 9b): a source phase of pH = 1 (nitric acid) and a concentration of 10^−2^ mol/L (1.39 g PNP/L), a receiving phase of pH = 11 (sodium hydroxide), a 4% NaNO_3_ electrolyte in aqueous phases and an *n*-alkyl alcohol membrane with 3 g/L of magnetic nanoparticles having a 1.12% silver content.

In the case of *n*-dodecanol, the results obtained indicated extraction efficiencies of at least 85% for the UDAc transporter and slightly lower, 82%, for the UDAl transporter for both target species. In the case of *n*-hexanol, the efficiency increased with the decrease of the number of carbon atoms in the alcohol molecule to a maximum of 99% in the case of the UDAc transporter and slightly lower, 95%, in the case of the UDAl transporter for both target species.

The extraction efficiencies were slightly lower for UDAl than for UDAc in all experimental situations, most likely due to UDAc’s acidity.

### 3.3. Study of the Influence of Aqueous Phase Parameters on the Performance of the Hybrid System

Under the experimental conditions imposed by the previously obtained results, it was possible to study the pH effect of the receiving phase over the transfer of the target chemical species through the 30-mm thick *n*-octanol liquid membrane containing 3 g/L of magnetic nanoparticles with a 1.12% silver composition, with 10–undecenoic acid (UDAc) and 10–undecylenyl alcohol (UDAl) as carriers, both with a concentration of 10^−3^ mol/L. The source phase had a neutral pH for both transported chemical species, with initial concentrations of 10^−3^ mol/L (SI) and 10^−2^ mol/L (PNP). The receiving phase had an acidic pH (HCL) for the transport of silver and a basic pH (NaOH) for the transport of *p*-nitrophenol. The flow rate of the source phase was 80 mL/min in all cases (through four drip holes), while the flow rate of the receiving phase was 20 mL/min (through only one drip hole), with the stirring speed of the magnetic nanoparticles being set to 100 rot/min.

**Figure 10:**
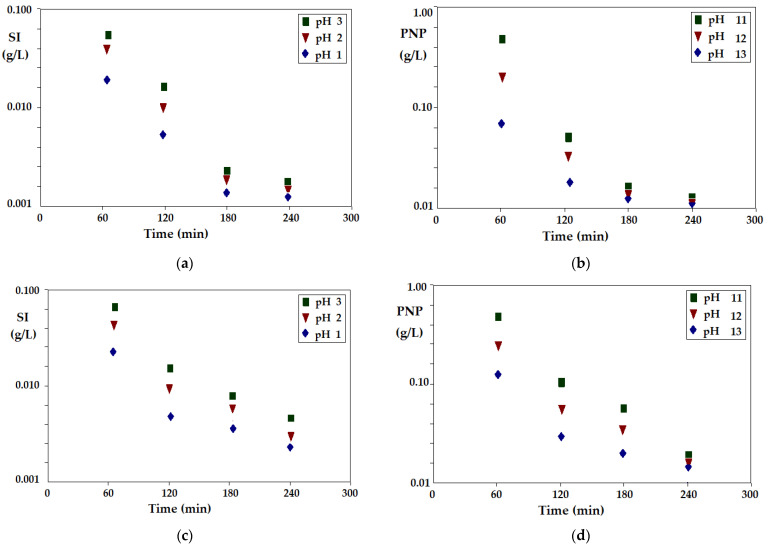
The decrease in the concentration of silver ions (SI) and *p*-nitrophenol (PNP), through an *n*-octanol membrane, depending on the pH of the receptor phase in the membrane phase, for a carrier of 10–undecenoic acid (UDAc), (**a**) and (**b**), respectively, and for carrier of 10–undecylenyl alcohol (UDA1), (**c**) and (**d**), respectively.

The results obtained (see Figure 10a–d) show that silver ions (SI) were transported faster if the pH of the receiving phase was lower, while *p*-nitrophenol (PNP) had a faster transport rate the higher was the pH of the receiving phase. The transport of chemical species was higher in the case of the UDAc carrier (Figure 10a,b) compared to the UDAl carrier (Figure 10c,d). The effect of the carrier change was more pronounced for silver ions than for *p*-nitrophenol.

**Figure 11:**
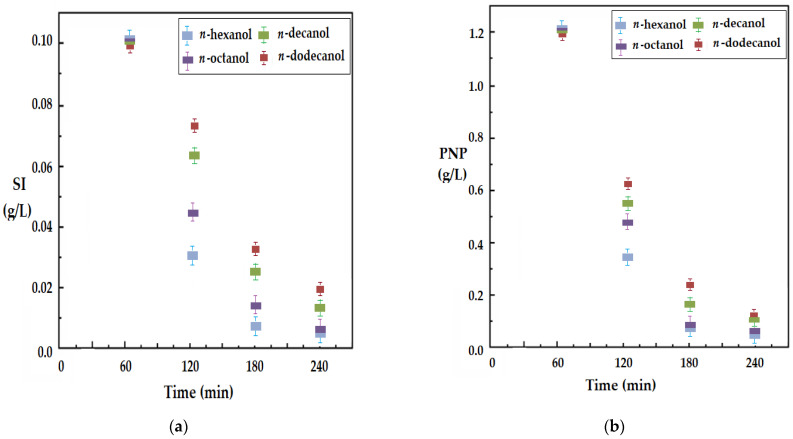
The decrease in the concentration, depending on time, of silver ions (SI) and *p*-nitrophenol (PNP) in the source phase through a membrane of *n*-alkyl alcohols with 6, 8, 10 and 12 carbon atoms, with a 10–undecenoic acid transporter (UDAc) and pH = 7 in the source phase, at the pH of the receiving phase: (**a**) pH = 1, for SI; (**b**) pH = 13, for PNP.

Figure 11 shows the results obtained under the same experimental conditions at the most favorable pH of the receiving phase for silver ions (Figure 11a) and for *p*-nitrophenol (Figure 11b) for all four working solvents, in the case of the UDAc carrier. The transport performance of both silver ions (SI) and *p*-nitrophenol (PNP) decreased with an increasing number of carbon atoms of the membrane solvent, most likely due to the viscosity of these alcohols (Table 1), and with the solubilization capacity of the transported species.

The observations made are in accordance with the literature that emphasizes the crucial importance of the carrier in the case of metal ion separation and the dominance of the properties of the membrane solvent in the case of molecular species [57,58,59,60].

**Figure 12:**
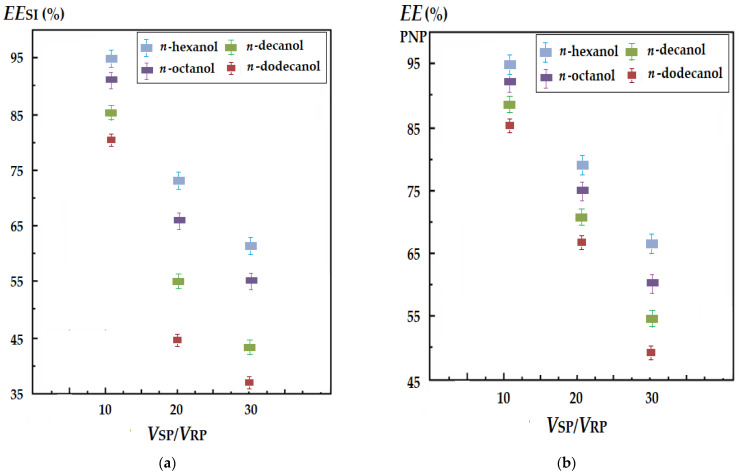
Efficiency of the separation of silver ions (SI) and *p*-nitrophenol (PNP) through a membrane of *n*-alkyl alcohols with 6, 8, 10 and 12 carbon atoms, having 10–undecenoic acid (UDAc) as a carrier, depending on the volume ratio of the source phase to the receiving phase: (**a**) for SI, pH = 1; (**b**) for PNP, pH = 13.

The study explored transporting and separating silver ions or *p*-nitrophenol from aqueous solutions. The volume of the source phase considered in experiments was 5000 mL, and the volume of the receiving phase was 500 mL, which would lead to separation efficiencies of over 90%, at a concentration factor (which represents the ratio between the concentration of the receiving phase and the concentration of the source phase at the end of the process) of at least 9 units. In order to establish the limit to which the volume of the source phase could be increased, at the same volume of 500 mL of the receiving phase, the efficiency of the extraction of the target chemical species was determined under the same working conditions. Doubling and tripling the volume of the source phase, respectively, were considered (Figure 12).

The efficiency of silver ion (SI) extraction at 3 h of operation decreased greatly as the ratio between the volumes of the source and receiver phases increased (Figure 12a), becoming more and more unfavorable with the increase of the number of carbon atoms in the membrane alcohol. Practically, at a ratio between the aqueous phases of 30 (15,000/500 mL), in the case of *n*-dodecanol, the extraction efficiency reached below 50%. The results recommend the use of *n*-hexanol and *n*-octanol up to a volume ratio of 20 (10,000/500 mL), when the extraction efficiency is still over 50%, even for *n*-decanol.

The efficiency of *p*-nitrophenol (PNP) extraction at 3 h of operation decreased more slowly as the ratio of the source and receiving phase volumes increased (Figure 12b). In this case, all four membrane solvents could be used, with extraction efficiencies that could be accepted at virtually all volume ratios. However, *n*-dodecanol must be the last one in the selection of the nature of the membrane phase, although it is very useful in terms of its loss in the aqueous phases.

### 3.4. Recommended Advantages, Limits and Parameters for the Proposed Hybrid Membrane System

The hybrid membrane system, proposed at this stage for laboratory experiments, combines the advantages of bulk liquid membranes (BLM) with those of emulsion membranes (ELM):

- Wide possibilities for varying the physical–chemical parameters of both the membrane and the aqueous phases;
- A large interphase transfer surface, ensuring convection both by means of source/receiving phase droplets passing through the membrane and through magnetic nanoparticles engaged by a rotating magnetic field (which can be achieved by electromagnetic means without moving elements);
- Easily adjustable recirculation rates of the aqueous phases;
- An easily adjusted thickness of the membrane;
- The volume of the source and receiving phase can be varied, and their ratio can be increased;
- It does not require surfactants to stabilize the drops;
- It does not require the breaking of an emulsion (the droplet size, imposed by the flow of the aqueous phases, is relatively large);
- Membrane solvents are biodegradable;
- The magnetic nanoparticles in the membrane phase can be promoters of turbulence but also carriers.

The proposed hybrid system can be physically made with accessible means: the body of the permeation module can be made of glass or polyethylene (a cylindrical vessel and a frustum funnel), a four-way peristaltic pump with an adjustable flow and flexible silicone rubber tubes (medical type or tourniquet).

On the other hand, some operating limitations or disadvantages must be emphasized:

- The membrane solvents are lost (at the solubility limit) in the aqueous phases;
- Special attention is needed to adjust the pH of the aqueous phases (a strong basic pH favors the appearance of emulsification and/or the increase in membrane solvent losses);
- The working temperature cannot be increased (because both the volatility and solubility of the membrane solvent will also increase);
- The flow at a single drip hole is limited and must be determined so that the drops are relatively large;
- The flow rate increases only by multiplying the drip holes;
- The volume of the membrane phase is still large, and the solvent losses in the aqueous phases are significant;
- The membrane solvents must be biodegradable.

## 4. Conclusions

This paper presents the operational limits of a type of hybrid membrane constituted as a bulk liquid membrane (BLM), but which works by dispersing the aqueous source phase (SP) and the receiving phase (RP), with the membrane itself being a dispersion of nanoparticles in an organic solvent (NP–OSM).

The main features and recommendations in operating the proposed experimental hybrid membrane system are a source phase volume of 5000–15,000 mL, a receiving phase volume of 500 mL, a membrane solvent volume of 300–500 mL, a volume ratio between the source and the receiving phase of 10–20:1, a volume ratio between the receiving phase and the membrane of 1–5:1, a membrane thickness of 30–40 mm, an aqueous phase flow through a single droplet mouth of 20 mL/min, an aqueous droplets’ diameter of 4–5 mm, *n*-octanol or *n*-decanol as the membrane solvent, magnetic nanoparticles (diameter of 40 nm ± 10 nm), ionic or molecular chemical species (silver ions and *p*-nitrophenol), a stirring speed of the magnetic rods of 100 rot/min and an operating temperature in the range 15–35 °C.

Under the exposed conditions, separation efficiencies of over 90% were obtained for both ionic and molecular chemical species (silver ions and *p*-nitrophenol).

The liquid membrane system with dispersed phases, presented in this paper, has superior results compared to the classical mechanically agitated liquid membrane systems (both the mass transfer surface and the convective transport increased). However, the proposed system does not cover the enormous mass transfer surfaces provided by emulsion membranes, but neither does it require specific methods for breaking the emulsions.

## Figures and Tables

**Table 1:** The characteristics of the liquid membranes components.

Component	Molar Mass (g/mol)	Density (830 kg/m^3^)	Solubility in Water (g/L)	Viscosity (cP)	Relative Polarity Measure *
*n*-hexanol	102.17	814	5.900	0.59	−0.579
*n*-octanol	130.23	830	0.300	7.36	−0.567
*n*-decanol	158.28	830	0.037	12.05	−0.540
*n*-dodecanol	186.34	831	0.004	18.80	−0.511
10–undecen–1–ol	170.29	846	0.044	–	–
10–undecylenic acid	184.28	912	0.074	–	–

* relative polarity measure.

**Table 2:** The used magnetic nanoparticles’ characteristics.

Nanoparticles	Composition Fe–Ag (%)		Medium Dimension (nm)	Saturation Magnetization (emu/g)	Refs.
NPFe–Ag1	70.59	0.21	35.4	1.40	[40]
NPFe–Ag2	60.86	0.93	38.7	1.31	[41,42]
NPFe–Ag3	57.55	1.12	41.5	1.13	[42]

**Table 3:** Bulk liquid membrane (BLM) and emulsion liquid membrane (ELM).

Liquid Membrane (LM)	Advantages	Disadvantages	Remedy Solutions
Bulk liquid membrane (BLM)	Accessible in the laboratory	High solvent consumption	Use of green solvents
	Adaptable design	Difficultly scaling	Development of process engineering
	Easy change of experimental conditions	Small mass transfer area	Development of new contact systems
Emulsion Liquid Membranes (ELM)	Huge mass transfer area	Emulsion instability	Development of process engineering
	Accessible pilot scaling	Use of surfactants	Use of biodegradable surfactants
	Wide possibilities to change the operational parameters	The need to recover the transported species by breaking the emulsion	Improvement of the ways of breaking emulsions

## Data Availability

Not applicable.
